# Influence of Normal Aging on Brain Autophagy: A Complex Scenario

**DOI:** 10.3389/fnagi.2019.00049

**Published:** 2019-03-11

**Authors:** David A. Loeffler

**Affiliations:** Beaumont Research Institute, Department of Neurology, Beaumont Health, Royal Oak, MI, United States

**Keywords:** aging, autophagy, brain, chaperone-mediated autophagy, macroautophagy

## Abstract

Misfolded proteins are pathological findings in some chronic neurodegenerative disorders including Alzheimer’s, Parkinson’s, and Huntington’s diseases. Aging is a major risk factor for these disorders, suggesting that the mechanisms responsible for clearing misfolded proteins from the brain, the ubiquitin-proteasome system and the autophagy-lysosomal pathway, may decline with age. Although autophagic mechanisms have been found to decrease with age in many experimental models, whether they do so in the brain is unclear. This review examines the literature with regard to age-associated changes in macroautophagy and chaperone-mediated autophagy (CMA) in the central nervous system (CNS). Beclin 1, LC3-II, and the LC3-II/LC3-I ratio have frequently been used to examine changes in macroautophagic activity, while lamp2a and HSPA8 (also known as hsc70) have been used to measure CMA activity. Three gene expression analyses found evidence for an age-related downregulation of macroautophagy in human brain, but no published studies were found of age-related changes in CMA in human brain, although cerebrospinal fluid concentrations of HSPA8 were reported to decrease with age. Most studies of age-related changes in brain autophagy in experimental animals have found age-related declines in macroautophagy, and macroautophagy is necessary for normal lifespan in *Caenorhabditis elegans, Drosophila*, and mice. However, the few studies of age-related changes in brain CMA in experimental animals have produced conflicting results. Investigations of the influence of aging on macroautophagy in experimental animals in systems other than the CNS have generally found an age-related decrease in Beclin 1, but conflicting results for LC3-II and the LC3-II/LC3-I ratio, while CMA decreases with age in most models. CONCLUSION: while indirect evidence suggests that brain autophagy may decrease with normal aging, this issue has not been investigated sufficiently, particularly in human brain. Measuring autophagic activity in the brain can be challenging because of differences in basal autophagic activity between experimental models, and the inability to include lysosomal inhibitors when measuring the LC3-II/LC3-I ratio in postmortem specimens. If autophagy does decrease in the brain with aging, then pharmacological interventions and/or lifestyle alterations to slow this decline could reduce the risk of developing age-related neurodegenerative disorders.

## Introduction

More than 30% of newly synthesized proteins are misfolded because of errors in translation or post-translational processes (Schubert et al., 2000). Molecular chaperones, primarily heat shock proteins, recognize these proteins and attempt to facilitate their refolding (Ciechanover and Kwon, 2017). Two proteolytic mechanisms, the ubiquitin-proteasome system (UPS) and the autophagy-lysosomal pathway (ALP), are responsible for cellular clearance of misfolded proteins which are unable to be refolded. The ALP includes three autophagic processes in mammals, namely macroautophagy, chaperone-mediated autophagy (CMA), and microautophagy (Orenstein and Cuervo, 2010), which use different mechanisms to deliver their cargo to lysosomes for enzymatic degradation. Misfolded proteins are initially subjected to removal by the UPS and selected proteins may also be removed by CMA. Misfolded proteins which avoid clearance by these mechanisms may be removed by macroautophagy (Ciechanover and Kwon, 2015). Protein oligomers, soluble aggregates, and intact organelles are too large to enter the proteasome’s narrow opening, so they can be degraded by the ALP but not by the UPS (Bence et al., 2001; Wu et al., 2015). Cross-talk is present between CMA and macroautophagy so that if one mechanism is impaired, the activity of the other may increase in an effort to maintain protein homeostasis (Massey et al., 2006a; Wu et al., 2015). Clearance of misfolded proteins is critical for cell survival because misfolding alters a protein’s three-dimensional structure, impairing its biological activities and increasing its propensity to form toxic aggregates (Hartl, 2017). Misfolded and aggregated proteins are key pathological findings in several chronic neurodegenerative diseases including Alzheimer’s disease (AD) (Selkoe, 2004), Parkinson’s disease (PD) (Tan et al., 2009), Huntington’s disease (Hatters, 2008), amyotrophic lateral sclerosis (Mulligan and Chakrabartty, 2013), and prion disorders (Kupfer et al., 2009); not surprisingly, dysregulation of the UPS and the ALP has been reported in some of these disorders (Bandyopadhyay and Cuervo, 2007; Wang et al., 2009; Alvarez-Erviti et al., 2010, 2013; Salminen et al., 2013; Murphy et al., 2015; Zheng et al., 2016; Budini et al., 2017; Guo et al., 2018). Because aging is the most consistent known risk factor for progressive neurodegenerative disorders (Jeppesen et al., 2011), the impairment of proteolytic mechanisms in these disorders may be due in part to an age effect (Lipinski et al., 2010; Xilouri and Stefanis, 2016).

The biological changes that occur in aging cells include alterations in proteostatic mechanisms, chromosome regulation, transcriptional regulation, protein translation, mitochondrial functioning, and cytoskeletal integrity (DiLoreto and Murphy, 2015). This review will focus on studies that have examined age-related changes in macroautophagy and CMA in the central nervous system (CNS) including the brain, cerebrospinal fluid (CSF), and retina. For comparative purposes, findings from studies which have investigated age-associated changes in these processes in non-CNS experimental systems will also be summarized.

## Autophagy Overview

**FIGURE 1:**
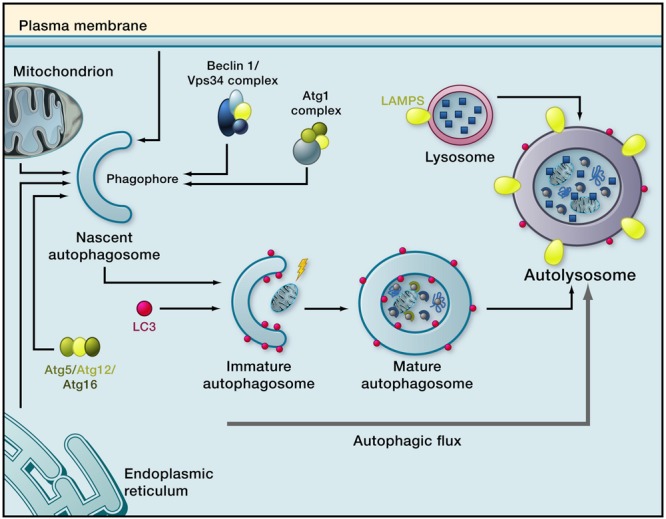
Macroautophagy. Induction of macroautophagy results in phagophore formation, which requires the kinase Vps34 (functioning in a complex that contains Atg6, whose mammalian homolog is Beclin 1). This is followed by phagophore membrane elongation, which is regulated by LC3-II. The phagophore closes around sequestered cargo, resulting in formation of a mature autophagosome. The autophagosome moves along microtubules to a lysosome and fuses with it. Cargo is then degraded by lysosomal enzymes and breakdown products are recycled to the cytosol. The complete process is termed autophagy flux (Reprinted from Rubinsztein et al., 2011).

Autophagy involves degradation of cytoplasmic contents within the lysosome, followed by recycling of the resulting macromolecular constituents including amino acids, sugars, lipids, and nucleic acids (Feng et al., 2014; Wu et al., 2015; Hewitt and Korolchuk, 2017). Macroautophagy (Figure 1), the best studied of these mechanisms, involves sequestering of cytoplasmic cargo into an autophagosome followed by lysosomal degradation of the cargo (Klionsky et al., 2016). A double-membraned structure, the phagophore, initially forms around bulk cytoplasm and its constituents (“non-selective macroautophagy”) or specific cytoplasmic targets including organelles (“selective macroautophagy”). The origin of the components which comprise the phagophore membrane is unclear; it has been suggested to derive from multiple sources including plasma membrane, endoplasmic reticulum, mitochondria, and the Golgi apparatus (Mari et al., 2011). Once autophagy is initiated, double-membrane nucleation allows formation of a phagophore which elongates, sequesters the cargo, and closes to form an autophagosome. The autophagosome then fuses with a lysosome to form an autolysosome (Seglen et al., 1990; Mizushima, 2007; Rubinsztein et al., 2011). Sequestered constituents are rapidly degraded by lysosomal hydrolytic enzymes. The term “autophagic flux,” which is a measure of autophagic degradation activity (Loos et al., 2014), is used to refer to the complete process which starts with autophagosome formation and ends with the release of macromolecules into the cytosol (Zhang et al., 2013). Quantification of autophagic flux requires multiple measurements of macroautophagy markers over time to determine the rate of the process (Loos et al., 2014).

Much of what is known about the induction of macroautophagy comes from studies with yeast, where 41 autophagy-related proteins, known as Atgs, have been identified to date (Noda and Mizushima, 2016; Pöggeler et al., 2018). Approximately half of these proteins are known to have homologs in higher eukaryotes (Klionsky and Codogno, 2013). The proteins necessary for autophagosome formation are referred to as “core molecular machinery” (Xie and Klionsky, 2007). The proteins involved in macroautophagy in mammals are similar but not identical to those in yeast; several reviews of the process in mammalian systems have appeared (Eskelinen, 2008; Mehrpour et al., 2010; Yang and Klionsky, 2010; Abounit et al., 2012). The core molecular machinery has been divided into functional groups (Yang and Klionsky, 2010; Noda and Mizushima, 2016): (a) the Atg1 kinase complex [in mammals, the Atg1/unc-51-like kinase (ULK) complex], (b) the Atg12-conjugation system, (c) the Atg8/microtubule-associated protein light chain 3 (LC3)-conjugation system, (d) the Vps34 phosphoinositide 3-kinase (PI3 kinase) complex, (e) the Atg9 and Atg2-Atg18 complex, and (f) vacuole-membrane-protein-1 (VMP1). ULK-1 and ULK-2 are mammalian proteins with close homology to Atg1. The Atg1/ULK complex, composed of ULK1 or ULK2, mAtg13 (the mammalian homolog of Atg13), Atg101, and FIP200 (the homolog of yeast Atg17), regulates macroautophagic activity by detecting changes in concentrations of constituents such as glucose, growth factors, nitrogen, amino acids, and reactive oxygen species (Kim et al., 2013). When nutrients are sufficient, mammalian TOR complex 1 (mTORC1) is included in this complex, but mTORC1 dissociates from it when nutrients are limiting. Macroautophagy can be induced by multiple mechanisms (Rubinsztein et al., 2011) including caloric restriction or treatment with rapamycin, both of which inhibit mTORC1. A decrease in mTOR’s kinase activity results in dephosphorylation of ULK1, ULK2, and mAtg13, which activates ULK1 and ULK2. ULK1 and ULK2 phosphorylate mAtg13 and FIP200, which initiates autophagosome formation (Hosokawa et al., 2009). Beclin 1, the mammalian homolog of Atg6, participates with other subunits in forming the PI3 kinase complex (Wu et al., 2015). The Atg12 and Atg8/LC3 conjugation systems are involved with elongation and expansion of the phagophore membrane (Yang and Klionsky, 2010). The Atg8-conjugation system conjugates Atg8 (and, in mammals, LC3) to phosphatidylethanolamine (PE). PE-conjugated LC3 is referred to as LC3-II, whereas its unconjugated counterpart is known as LC3-I. LC3-II regulates phagophore membrane elongation (Abeliovich et al., 2000) and facilitates membrane tethering and hemifusion (Nakatogawa et al., 2007). The Vps34 PI3 kinase complex is a positive regulator of macroautophagy; its activity generates phosphatidylinositol 3-phosphate, which recruits the Atg2–Atg18 complex to bind to Atg9 on autophagic membranes (Obara and Ohsumi, 2008). Atg9 also carries membranes from donor organelles between the developing autophagosome and the periphery. VMP1, a mammalian protein which does not appear to have an equivalent in yeast, induces autophagy even when nutrients are adequate by binding to Beclin 1, leading to the formation of a complex with Vps34 (Molejon et al., 2013). UV radiation-associated resistance gene (UVRAG) and Beclin 1-associated autophagy related key regulator (Barkor) stabilize the Beclin 1/Vps34 complex (Liang et al., 2008; Sun et al., 2008). Other important regulators of macroautophagy include AMP-activated protein kinase (AMPK) (Meley et al., 2006) and the PI3K/Akt/mTOR pathway (Woo et al., 2017). AMPK is activated by low ATP or glucose concentrations. Its activation can induce macroautophagy by inactivating TORC1 and by phosphorylating ULK1 (Hardie, 2011). Conversely, signaling from the PI3K/Akt/mTOR complex leads to phosphorylation, and thus activation, of mTOR (Triplett et al., 2015), which inhibits macroautophagy (Cao et al., 2014).

Markers which have been used to measure macroautophagy (reviewed by Klionsky et al., 2016) include the proteins encoded by Atg5 (Codogno and Meijer, 2006) and Atg12 (Hanada and Ohsumi, 2005), Beclin 1 (Kang et al., 2011), mTOR (Brown et al., 1994), and LC3 (Sugawara et al., 2004), including LC3-I, LC3-II, and the LC3-II/LC3-I ratio. LC3-II is initially found on inner and outer layers of the phagophore membrane. During autophagosome maturation, it is removed from the outer (cytosolic) surface due to cleavage of PE by Atg4 (Yu et al., 2012), but remains on the inner (lumenal) surface of the autophagosome. LC3-II levels therefore correlate with autophagosome numbers (Mizushima and Yoshimori, 2007; Wu et al., 2015). The LC3-II/LC3-I ratio is often examined by Western blot at a single time point to assess changes in macroautophagic activity, but this procedure does not actually measure autophagic flux because no rate of change is measured (Loos et al., 2014). Alterations in LC3-II and in the LC3-II/LC3-I ratio can be difficult to interpret, because an increase in the LC3-II level may be due to either activation of macroautophagy or a decrease in autophagosomal-lysosomal fusion or lysosomal degradation of cargo; similarly, a reduction in LC3-II may be due to inhibition of macroautophagy or to an increase in autophagic flux (Linton et al., 2015). Preventing lysosomal degradation of autophagosomal LC3-II, by including inhibitors of autophagosome-lysosome fusion or of lysosomal proteases in the experimental system, may be necessary to interpret LC3-II and LC3-II/LC3-I measurements (Klionsky et al., 2016; Orhon and Reggiori, 2017). Other markers that have been used to investigate changes in macroautophagy include the lysosomal protease cathepsin D (Tatti et al., 2012) and the autophagic adaptor proteins p62/Sequestosome-1 (known as p62/SQSTM1 or p62) (Lippai and Löw, 2014) and its functional homolog, neighbor of BRCA1 (NBR1) (Kirkin et al., 2009). The latter two proteins are macroautophagy substrates; however, similar to the situation with LC3-II, single measurements of these proteins provides no information about their rate of turnover (Loos et al., 2014).

**FIGURE 2:**
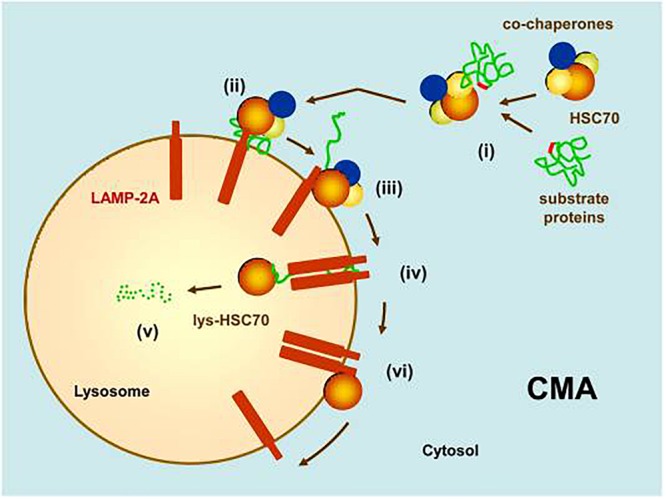
Chaperone-mediated autophagy (CMA). (i) Substrate proteins with an amino acid sequence biochemically similar to KFERQ are recognized by HSPA8/hsc70 in association with co-chaperones. (ii) This complex binds to the monomeric form of a lysosomal surface receptor, lamp2a, which will facilitate its translocation into the lumen of the lysosome. (iii) Binding of the substrate to lamp2a causes lamp2a to multimerize into a translocation complex. (iv) After unfolding, the substrate translocates into the lysosomal lumen. The presence of a lysosomal luminal form of HSPA8/hsc70 is required for this translocation to occur. (v) The substrate is quickly degraded by lysosomal hydrolytic enzymes. (vi) Lamp2a disassembles from its translocation complex, facilitated by HSPA8/hsc70. (Reprinted from Cuervo, 2010).

Chaperone-mediated autophagy (Figure 2), a more selective autophagic mechanism than macroautophagy which has been identified only in mammals, does not involve vesicle formation (Cuervo, 2010). Its selectivity is due to the requirement for its substrate proteins to contain amino acid sequences biochemically similar to Lys-Phe-Glu-Arg-Gln (KFERQ), which is present in approximately 30% of cytosolic proteins (Dice et al., 1986; Chiang and Dice, 1988; Chiang et al., 1989). Proteins bearing this sequence are bound by the molecular chaperone heat shock 70 kDa protein 8 (HSPA8, also known as hsc70 and hsc73; hereafter, “HSPA8/hsc70”) in association with co-chaperones (Agarraberes and Dice, 2001). The protein-HSPA8/hsc70 complex then binds to a lysosomal surface receptor, lysosome-associated membrane glycoprotein 2a (lamp2a), which facilitates its translocation into the lysosomal lumen. Binding of the protein-HSPA8/hsc70 complex to lamp2a is rate-limiting for CMA (Cuervo and Dice, 1996) so the concentration of lamp2a on lysosomal membranes is considered to be a marker for CMA (Cuervo and Dice, 2000b; Patel and Cuervo, 2015). In CMA, substrate proteins must unfold before their translocation into the lysosomal lumen. This unfolding is facilitated by HSPA8/hsc70 and its co-chaperones (Salvador et al., 2000). Only lysosomes containing HSPA8/hsc70 on the lumenal side of the lysosomal membrane are capable of performing CMA (Patel and Cuervo, 2015). The percentage of CMA-competent lysosomes in the rat liver is 20–30% under basal conditions but can increase to 80% during starvation (Cuervo et al., 1997).

Microautophagy has been less studied than the other autophagic mechanisms. In this process the lysosomal membrane directly engulfs portions of cytoplasm and the constituents within it. This process can be non-selective (degradation of randomly sequestered cytosol) or selective (degradation of specific organelles such as mitochondria, portions of the nucleus, or peroxisomes) (Farré et al., 2009; Mijaljica et al., 2011). Little is known about the factors that regulate microautophagy in mammalian cells (Mijaljica et al., 2011).

## Cross-Talk Between Autophagic Mechanisms

**FIGURE 3:**
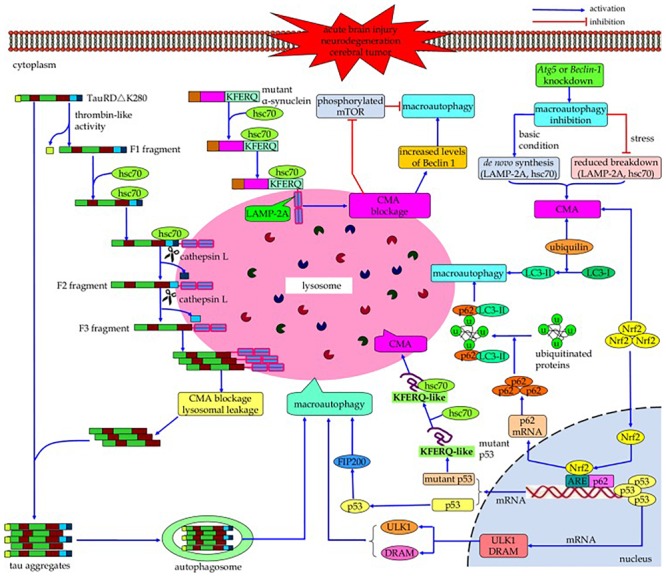
Cross-talk between macroautophagy and CMA. If the activity of one of these autophagic processes is reduced, the activity of the other may increase in a compensatory manner. Continued blockage of CMA by inhibitory RNA targeting of lamp2a results in activation of macroautophagy as indicated by increased levels of Beclin 1; conversely, while nutrient deprivation initially upregulates macroautophagy, continued nutrient deprivation downregulates it with compensatory activation of CMA. However, this compensation may not be bi-directional, and the upregulation of one mechanism may not fully compensate for impairment of the other one. The signaling mechanisms involved in this cross-talk, while poorly understood, may include HSPA8/hsc70, p53, Nrf2, and/or ubiquilin (Reprinted from Wu et al., 2015).

Both CMA and macroautophagy are maximally activated by stress conditions such as starvation and oxidative stress (Kaushik et al., 2008). Cross-talk occurs between these two autophagic mechanisms (Figure 3) (reviewed by Wu et al., 2015); if the activity of one is reduced, the activity of the other may increase in an effort to maintain protein homeostasis (Cuervo et al., 2004; Massey et al., 2006a,b, 2008; Kaushik et al., 2008; Wang et al., 2010). For example, while macroautophagy is initially upregulated due to nutrient deprivation, if this condition continues it becomes downregulated, with compensatory activation of CMA (Cuervo et al., 1995; Massey et al., 2006a). Conversely, blockage of CMA by inhibitory RNA targeting of lamp2a initially results in a decline in macroautophagy due to increased mTOR activity, but if CMA blockage continues then macroautophagy becomes activated as indicated by increased levels of Beclin 1. In a cell model of tauopathy, failure of CMA to clear mutated tau led to formation of tau aggregates, which could be cleared by macroautophagy (Wang et al., 2010). The signaling mechanisms in this cross-talk are incompletely understood; proteins suggested by Wu et al. (2015) to be involved in this process include HSPA8/hsc70 (Vakifahmetoglu-Norberg et al., 2013), p53 (Tasdemir et al., 2008), nuclear factor (erythroid-derived 2)-like 2 (Nrf2) (Gan et al., 2012), and ubiquilin (Rothenberg et al., 2010). Reduced phosphorylation of mTOR may play a role in the compensatory increase in macroautophagy when CMA is impaired (Massey et al., 2008), and when macroautophagy is inhibited, compensatory upregulation of CMA can occur through increasing lysosomal concentrations of lamp2a and HSPA8/hsc70 (Kaushik et al., 2008; Koga et al., 2011). Upregulation of CMA may not fully compensate for impairment in macroautophagy, and vice versa (Massey et al., 2006b; Wu et al., 2015). The cross-talk between the autophagic processes is not always bidirectional. Rodríguez-Muela et al. (2013) found an age-associated decrease in macroautophagy (decreased LC3-II/LC3-I ratio and reduced mRNA expression of Beclin 1 and Atg7) in the mouse retina, which was accompanied by an increase in CMA (increased lysosomal levels of lamp2a and HSPA8/hsc70). A similar result was obtained *in vitro* by shRNA-mediated knockdown of Atg7 expression in the 661W mouse retinal cell line. However, inhibition of CMA in these cells (by downregulating lamp2a) did not result in an increase in macroautophagy. The interactions between CMA and macroautophagy have been suggested to delay the accumulation of abnormal proteins, perhaps contributing to the association between aging and neurodegenerative disorders (Cuervo and Wong, 2014).

## Influence of Normal Aging on Autophagy in Human Brain and CSF

Few studies have been performed on the effects of normal aging on autophagy in human brain or CSF. The findings in these studies are summarized in Table 1. Three gene expression studies with human brain have found age-associated reductions in macroautophagy markers. Shibata et al. (2006) found downregulation of Beclin 1, and Lipinski et al. (2010) found decreases in autophagy-regulating genes including Atg5 and Atg7. Guebel and Torres (2016), investigating the effects of gender and aging on gene transcription in the hippocampus, reported decreased expression of LC3, HDAC6 (a deacetylase required for autophagosomal maturation and fusion with lysosomes) (Lee J.H. et al., 2010), and PINK1 (a mitochondrial kinase whose activity is crucial to mitochondrial function) (Qu et al., 2015) in older women. In older men expression of Bcl-2, which inhibits Beclin 1 (Liang et al., 1999), was increased, suggesting a decrease in macroautophagic activity. Conversely, expression of BAG-2, which inhibits PINK1 degradation by blocking its ubiquitination, and therefore triggers PARKIN-mediated mitophagy (Qu et al., 2015), was also increased in older men, suggesting activation of mitophagy. Notably, no studies were found of the effects of normal aging on CMA activity in human brain.

**Table 1:** Influence of normal aging on autophagy in human brain and CSF.

Study	Results
Shibata et al., 2006	Beclin 1 expression decreased in age-dependent fashion in human brains.
Lipinski et al., 2010	Genome-wide analysis found transcriptional downregulation of key macroautophagy genes including Atg5 and Atg7 in older (>70 years old) vs. younger (<40 years old) human brains.
Baird et al., 2012	Proteomics study on CSF from cognitively normal adults; three proteins in autophagy category of gene ontological analysis were positively associated with age.
Guebel and Torres, 2016	Influence of sexual dimorphism and aging on gene transcription in hippocampus: expression of LC3 and HDAC6 decreased in older women; in older men, expression of bcl-2, which inhibits Beclin 1, was increased. However, BAG-2, which can trigger PARKIN-mediated mitophagy, also increased in older men.
Loeffler et al., 2016	HSPA8/hsc70 in CSF from healthy subjects was negatively correlated with age.
Loeffler et al., 2018	Lamp2 concentration in CSF changed little during normal aging.

*Three gene expression studies on human postmortem brain specimens have found age-associated decreases in macroautophagy markers. No studies of the effects of normal aging on CMA activity in human brain were found. Three studies of age-related changes in CSF autophagy proteins have produced conflicting results*.

Three studies have examined the effects of normal aging on autophagy-related proteins in CSF. A proteomics analysis found CSF concentrations of three proteins with the gene ontology classification of “autophagy,” namely myoglobin, MMP8, and HMW kininogen, to be positively correlated with age (Baird et al., 2012). Conversely, the CSF concentration of HSPA8/hsc70 was found to decrease with age (Loeffler et al., 2016) while the CSF level of lamp2 changed little (Loeffler et al., 2018). Lamp2 is not a marker for a specific autophagic process, because in addition to the previously discussed role of lamp2a in CMA (Cuervo and Dice, 2000a), lamp2 has two other isoforms which also participate in autophagic processes (Nishino et al., 2000; Fujiwara et al., 2015).

## Influence of Normal Aging on Autophagy in the CNS in Experimental Models

Studies of the relationship between aging and brain macroautophagy, including age-related changes in macroautophagy markers and the effects of impairing macroautophagy (for example, by introducing mutations in Atg genes) on lifespan, are shown in Table 2 for experimental animal models. This table is not all-inclusive; additional references can be found in recent reviews by Hansen et al. (2018) and Nakamura and Yoshimori (2018). Macroautophagy has been shown to be required for normal lifespan in *Caenorhabditis elegans* and *Drosophila* (Meléndez et al., 2003; Hars et al., 2007; Simonsen et al., 2008; Minnerly et al., 2017) and enhancement of macroautophagic activity, for example by upregulation of AMPK or rapamycin-mediated inhibition of TOR, increases lifespan in these models (Vellai et al., 2003; Simonsen et al., 2008; Bjedov et al., 2010; Ulgherait et al., 2014). However, conflicting results were found by Hashimoto et al. (2009), who reported that knockout of some autophagy genes in daf-2 mutant *C. elegans* mutants increased rather than decreased their lifespan. Also in C. elegans, Saha et al. (2015) found an age-associated loss of macroautophagic function in dopamine neurons. A recent review by Nakamura and Yoshimori (2018) concluded that “basal level of autophagic activity is elevated in many longevity paradigms and the activity is required for lifespan extension.” The effects of aging on brain macroautophagy have also been investigated in mice (Hara et al., 2006; Komatsu et al., 2006; Gamerdinger et al., 2009; Kaushik et al., 2012; Triplett et al., 2015; Ott et al., 2016), rats (Yu et al., 2017), and cows (De Biase et al., 2017). Mice lacking Atg7 in the CNS developed neuronal loss and died within 28 weeks (Komatsu et al., 2006), and mice deficient for Atg5 in neural cells developed progressive motor deficits in conjunction with neuronal inclusion bodies (Hara et al., 2006). An association was reported between activation of macroautophagy and retention of spatial memory during aging; mice that were subjected to caloric restriction for 10 months, beginning at 6 weeks of age, performed better on Morris water maze testing than mice fed a high calorie diet, and the mice subjected to caloric restriction had higher hippocampal levels of Beclin 1, LC3, and cathepsin B and lower levels of mTOR and p62 than the high-calorie group (Dong et al., 2015). Kaushik et al. (2012) and Ott et al. (2016) reported age-related decreases in macroautophagy markers in mouse brain, and similar findings were reported by Yu et al. (2017) in rat brain. Brain levels of Beclin 1 were found to decline in old naked mole-rats, the longest-lived rodent, suggesting an age-associated decrease in macroautophagy in this species as well (Triplett et al., 2015). Rodríguez-Muela et al. (2013), examining age-related changes in autophagic mechanisms in mouse retina, found lower Beclin 1, and lower LC3-II after lysosomal blockage, in older mice, and De Biase et al. (2017) found lower Beclin 1 but an increase in the LC3-II/LC3-I ratio in old cow brain specimens. In conflict with these results is a study by Gamerdinger et al. (2009) which reported increased total cathepsin and cathepsin B activity in old mouse brain. In that study, aging was associated with a switch of regulatory control over degradation of polyubiquitinated proteins in mouse brain from Bcl-2-associated athanogene-1 (BAG1), a regulator of proteasomal degradation pathways (Lüders et al., 2000), to BAG3, a stimulator of macroautophagy (Carra et al., 2008), suggesting that aged cells may rely more on macroautophagy for degrading polyubiquitinated proteins.

**Table 2:** Influence of normal aging on CNS macroautophagy in experimental systems.

Study	Results
Meléndez et al., 2003	Knockdown of bec-1 (*C. elegans* homolog of mammalian APG6/VPS30/beclin1) prevented lifespan extension in *daf-2 C. elegans* mutants.
Vellai et al., 2003	TOR deficiency in *C. elegans* increases its lifespan.
Hara et al., 2006	Mice deficient for Atg5 in neural cells developed progressive motor deficits together with neuronal inclusion bodies.
Komatsu et al., 2006	Mice lacking Atg7 in the CNS developed behavioral defects and died within 28 weeks. Neuronal loss was present in cerebral and cerebellar cortices, with age-related accumulation of polyubiquitinated proteins as neuronal inclusion bodies.
Hars et al., 2007	Knockdown of atg7 and atg12 shortened the lifespan of both wild type and insulin/IGF-1 receptor *daf-2* mutant *C. elegans*.
Simonsen et al., 2008	Expression of several autophagy genes in *Drosophila* neural tissues decreases with aging. Atg8a mutations reduce lifespan and increase sensitivity to oxidative stress.
Hashimoto et al., 2009	Knockout of macroautophagy genes tended to reduce lifespan in wild type *C. elegans*, but knockout of any of seven autophagy genes in *daf-2 C. elegans* mutants increased lifespan.
Gamerdinger et al., 2009	Increased BAG-3 (regulator of macroautophagy) and decreased BAG-1 (regulator of proteasomal pathways) were found in brain regions of old mice.
Bjedov et al., 2010	Inhibition of TOR by rapamycin increased lifespan in *Drosophila*.
Kaushik et al., 2012	22-month-old mice had lower levels of Atg7, LC3-II, p62, and NBR1 in hypothalamus than 3-month-old mice.
Rodríguez-Muela et al., 2013	No differences in basal levels of LC3-II in retinas between 3- and 22-month-old mice, but in contrast to findings in young mice, no increase in LC3-II occurred in the old mice after lysosomal blockage. mRNA for beclin-1 and Atg7 was decreased in the older animals, and protein levels of the macroautophagy substrate p62 were increased.
Dong et al., 2015	An association was found between induction of macroautophagy activity (by caloric restriction) and retention of spatial memory during aging in mice. Caloric restriction resulted in higher hippocampal levels of Beclin 1, total LC3, and cathepsin B and lower levels of mTOR and p62.
Saha et al., 2015	Age-related inhibition of macroautophagy in dopaminergic neurons after *C. elegans* finished reproductive period.
Ott et al., 2016	Beclin 1, p62, and ATG5-ATG12 decreased with age, and mTOR increased with age, in C57BL6 mouse brain.
Triplett et al., 2015	Beclin 1 decreased in whole-brain homogenates from old naked mole-rats, while the LC3-II/LC3-I ratio did not change with age.
De Biase et al., 2017	Old cow brain specimens had higher LC3 immunoreactivity, lower Beclin 1 immunoreactivity, and increased LC3-II/LC3-I ratio compared to young cow brain specimens.
Minnerly et al., 2017	A mutation in the atg-18 gene reduces *C. elegans* lifespan. Expression of atg-18 in neurons or intestinal cells restores normal lifespan, with similar results in *daf-2* mutants.
Yu et al., 2017	24-month old Wistar rats had lower hippocampal Beclin 1 and LC3-II than 5-month-old rats.

*Most studies in *C. elegans* and *Drosophila* have reported decreased brain macroautophagy with aging. Macroautophagy has been shown to be required for normal lifespan in these models. In mouse brain, macroautophagy markers decrease with aging. Knockout of critical Atgs results in neurodegeneration and shortened lifespan in mice*.

Few studies have investigated the effects of normal aging on CMA in the CNS in experimental models. The results from these studies are summarized in Table 3. Three studies examined the influence of normal aging on the concentration of HSPA8/hsc70 in rat brain. Two of these reported an increase (Unno et al., 2000; Calabrese et al., 2004) while the third one found a decrease (Gleixner et al., 2014). In the study discussed above in which an age-related decrease in macroautophagy was found in mouse retina (Rodríguez-Muela et al., 2013), CMA was reported to increase with normal aging, possibly to compensate for the decrease in macroautophagy.

**Table 3:** Effects of normal aging on CNS CMA in experimental systems.

Study	Results
Unno et al., 2000	HSPA8/hsc70 in pons, medulla, striatum, and thalamus was higher in 24- than in 6-month-old Wistar rats.
Calabrese et al., 2004	Age-related increase in HSPA8/hsc70 in rat brain between 6 and 28 months of age; highest levels in hippocampus and substantia nigra, followed by cerebellum, cortex, septum and striatum.
Rodríguez-Muela et al., 2013	An age-related increase was found in lamp2a and HSPA8/hsc70 concentrations in mouse retina, possibly to compensate for the age-related decrease in macroautophagy. However, in cultured cone retinal cells, CMA blockade did not result in an increase in macroautophagy.
Gleixner et al., 2014	HSPA8/hsc70 was lower in striatum, but not in substantia nigra, of old vs. young female rats.

*Few studies have been performed to investigate the effects of normal aging on CMA in the CNS in experimental animal models. Two studies have reported an age-related increase in HSPA8/hsc70 in rat brain while a third one found a decrease. In mouse retina, an age-related increase in CMA was detected, possibly in compensation for an age-related decrease in macroautophagy*.

## Influence of Normal Aging on Autophagy in Experimental Systems Other Than the CNS

The experimental models that have been used most frequently to examine the effects of aging on macroautophagy in non-CNS systems are human fibroblast cultures (Tashiro et al., 2014; Ott et al., 2016; Pernodet et al., 2016; Romero et al., 2016) and rat liver, in which Bergamini and colleagues have extensively investigated the effects of caloric restriction on macroautophagy and its endocrine regulation (Vittorini et al., 1999; Cavallini et al., 2001, 2007; Donati et al., 2001a,b, 2008, 2009; Del Roso et al., 2003). Studies have also been performed in mouse liver (Terman, 1995), rat heart (Wohlgemuth et al., 2007), mouse muscle (Masiero et al., 2009; Carnio et al., 2014), mouse heart (Inuzuka et al., 2009; Taneike et al., 2010; Boyle et al., 2011; Zhou et al., 2017), rat nucleus pulposis (Ye et al., 2011), and human muscle biopsies (Carnio et al., 2014). The results in these studies are summarized in Table 4. Most studies using Beclin 1 as a marker for macroautophagy have found an age-related decrease, while conflicting results have been obtained with measurements of LC3-II and LC3-II/LC3-I.

**Table 4:** Influence of normal aging on macroautophagy in experimental systems other than the CNS.

Study	Results
Terman, 1995	Decreased rate of formation of autophagic vacuoles, and decreased rate of their elimination, in hepatocytes from old (20–21 months) CBA mice.
Vittorini et al., 1999	Reduced hepatic macroautophagy in old Sprague-Dawley rats, partially prevented by dietary restriction.
Donati et al., 2001a	Maximum rate of autophagic proteolysis reached at 6 months in Sprague-Dawley rats and declined thereafter.
Donati et al., 2001b	Autophagy in isolated liver cells of Sprague-Dawley rats exhibited age-related decline after 6 months.
Del Roso et al., 2003	Autophagic-proteolytic response of liver to an anti-lipolytic agent was maximal in 1-month-old Sprague-Dawley rats, decreased in 6-month-old rats, and almost negligible in older rats.
Cavallini et al., 2007	Oxidized mitochondria accumulated in older Sprague-Dawley rat liver rather than degradation by autophagy.
Wohlgemuth et al., 2007	Beclin 1 increased in heart from 26-month old vs. 6-month old Fisher 344 rats.
Donati et al., 2008	Autophagic response to glucagon and insulin decreased in isolated hepatocytes from older rats.
Ye et al., 2011	Increased LC3-II and LC3-II/LC3-I ratio in nucleus pulposis of 24 month-old vs. 3 month-old Sprague-Dawley rats.
Carnio et al., 2014	Age-associated decrease in LC3-II and Atg7 in muscle from older humans and mice.
Tashiro et al., 2014	Increased autophagosome number and LC3-II, but no change in Beclin 1 or Atg5, in skin fibroblasts from older women.
Ott et al., 2016	Decreased LC3-II/LC3-I ratio, p62, ATG5–ATG12, and beclin-1 in older (60 doublings) vs. younger (20 doublings) human dermal fibroblasts; higher mTOR (macroautophagy inhibitor) in old fibroblasts.
Pernodet et al., 2016	Reduced mRNA expression of LC3-II in synchronized aged normal human skin fibroblasts compared to young skin fibroblasts.
Romero et al., 2016	Reduced induction of autophagy in aged human primary lung fibroblasts.
Zhou et al., 2017	Decreased autophagic degradation of p62 in skeletal and cardiac muscle in 27-month old vs. 5-month old mice, and decreased LC3-II/LC3-I ratio in skeletal muscle from aged mice.

*Many studies have been performed to examine the effects of aging on macroautophagy in rat liver and human fibroblasts. Most studies have found an age-related decrease.*

Studies on the effects of aging on CMA in experimental systems other than the CNS are summarized in Table 5. The initial studies on CMA were done by Dice and his colleagues (Dice, 1982; Okada and Dice, 1984; McElligott et al., 1985; Dice et al., 1986; Chiang and Dice, 1988; Chiang et al., 1989; Cuervo and Dice, 1996). Cuervo and Dice (2000a) reported that CMA decreased in lysosomes from aged rat livers and late-passage human fibroblasts, in association with an age-associated decrease in lamp2a on lysosomal membranes. Their finding of an age-related decrease in CMA in rat liver has been confirmed and extended in subsequent studies (Kaushik et al., 2007; Kiffin et al., 2007; Zhang and Cuervo, 2008; Bandyopadhyay et al., 2010; Rodríguez-Navarro and Cuervo, 2012; Schneider et al., 2015). Age-related changes in CMA have also been studied in mouse skeletal and cardiac muscle (Zhou et al., 2017) and rat nucleus pulposis (Ye et al., 2011), with conflicting results. An age-associated decrease in lamp2 gene expression was reported for human leukocytes (Huang et al., 2012) but as discussed above, lamp2 measurements are not specific for CMA.

**Table 5:** Effects of normal aging on CMA in experimental systems other than the CNS.

Study	Results
Okada and Dice, 1984	Reduced ability of late-passage confluent human fibroblasts to degrade long-lived, but not short-lived, proteins in absence of growth factors.
Cuervo and Dice, 2000a	CMA decreased in lysosomal membranes from aged (22 month-old) vs. young (3 month-old) rat liver, and in higher-passage (52 population doublings) vs. lower passage (22 population doublings) human fibroblasts. Age-related reduction in lamp2a at lysosomal membrane in both systems. Increased hsc73 (HSPA8/hsc70) at hepatic lysosomal membrane in old rats; may be compensatory response to age-related reduction in CMA.
Kiffin et al., 2007	Age-related decline in CMA in rat liver due to altered dynamics and stability of lamp2a at lysosomal membrane. Rate of transcription of lamp2a unchanged with age.
Kaushik et al., 2007	Age-related changes in lipid composition of discrete microdomains at the lysosomal membrane found to be responsible for reduced lysosomal levels of lamp2a with aging in rat hepatocytes.
Zhang and Cuervo, 2008	Age-related decline in CMA prevented in transgenic mouse in which lysosomal membrane lamp2a concentration was modulated. Preservation of autophagic activity resulted in reduced intracellular accumulation of damaged proteins.
Ye et al., 2011	Increased lamp2a, but decreased HSPA8/hsc70, in nucleus pulposis of 24 month-old vs. 3 month-old Sprague-Dawley rats.
Huang et al., 2012	Age-related decrease in lamp2 gene expression in human leukocytes.
Rodríguez-Navarro et al., 2012	Chronic exposure to high-fat diet or acute exposure to cholesterol-enriched diet decreased hepatic CMA in mice due to lower lamp2a at lysosomal membrane. Changes in lipid composition of the lysosomal membrane of lipid-challenged animals were similar to changes caused by aging.
Schneider et al., 2015	In a mouse model with liver-specific CMA deficits, other proteolytic systems were able to compensate for these deficits in young mice but not in older mice.
Zhou et al., 2017	Lamp2a and HSPA8/hsc70 decreased in skeletal muscle of aged (27 months old) vs. young (5 months old) C57BL/6 mice; in cardiac muscle of aged mice, lamp2a increased while HSPA8/hsc70 was unchanged.

*Most studies have examined this issue in rat liver, and have reported an age-related decrease in CMA. Similar findings were reported for late-passage human fibroblasts, while conflicting results have been found for mouse skeletal and cardiac muscle and rat nucleus pulposis*.

## Influence of Normal Aging on Lysosomal Activity

Normal aging has been associated with decreased lysosomal activity (Reeg and Grune, 2015; Carmona-Gutierrez et al., 2016). Successful autophagic removal of proteins requires an efficiently functioning lysosomal pool, so a decline in lysosomal activity could play a prominent role in age-related deficits in macroautophagy and CMA. Lysosomal hydrolytic enzymes include proteases, nucleases, lipases, sulfatases, and phosphatases, whose optimal pH range is 4.5–5 (Carmona-Gutierrez et al., 2016). In yeast, lysosomal pH is a regulator of lifespan and mitochondrial function (Hughes and Gottschling, 2012; Ruckenstuhl et al., 2014). Lysosome numbers and their size increase in senescent cells (Robbins et al., 1970; Brunk et al., 1973; Kurz et al., 2000), possibly due to accumulation of non-degradable constituents such as lipofuscin in autophagic vacuoles (Brunk and Terman, 1999). Lipofuscin accumulates during normal aging in lysosomes of neurons and other post-mitotic cells, where it may exert deleterious effects on autophagy if lysosomal enzymatic activity is expended in an effort to degrade it (Brunk and Terman, 2002). The literature contains conflicting results with regard to age-related changes in the activities of different lysosomal proteases in the CNS. Kenessey et al. (1989) and Nakamura et al. (1989) reported that cathepsin D activity increased in the aged rat brain, and Nakanishi et al. (1994) found increases in cathepsin D, E, and B activities, but decreased cathepsin L activity (although not in cathepsin L protein), in brain regions from older rats. Cathepsin D localized primarily to lysosomes in young rat cerebral cortical neurons, but diffuse cytosolic immunoreactivity was also present in older rats, possibly contributing to age-related cell death (Jung et al., 2010). (See Stoka et al., 2016 for a review of the regulation of lysosomal cathepsins in aging and neurodegeneration and their contribution to apoptotic cell death.) Conversely, Sarkis et al. (1988) reported age-related decreases in the activities of cathepsins D, Ce1, and Ce2 in *C. elegans*. Finally, Banay-Schwartz et al. (1992), measuring cathepsin D in 50 brain regions of adult and older individuals, found the levels of this enzyme to be significantly increased with age in 14 regions and decreased in two regions. The findings in these studies suggest that an age-related decline in lysosomal function is not necessarily accompanied by decreases in the activities of lysosomal proteases.

## Discussion and Conclusion

Autophagic processes have often been stated to decrease with age (Martinez-Lopez et al., 2005; Martinez-Vicente et al., 2005; Cuervo, 2008; He et al., 2013; Cuervo and Wong, 2014; Schroeder et al., 2014). This conclusion is supported by most studies that have examined this issue in experimental systems other than the CNS (Tables 4, 5). The literature also suggests an age-related decline in macroautophagy in the CNS in experimental models, particularly *C. elegans, Drosophila*, and mice (Table 2). But because of the few studies on this issue in the human CNS (Table 1), no conclusion is currently possible as to whether autophagic processes in human brain decrease with normal aging, despite indirect evidence for this possibility from the studies in experimental systems. A similar conclusion was reached in a recent review by Hansen et al. (2018) of the connection between macroautophagy and longevity: “…further work is required to more rigorously test the hypothesis that autophagic activity may decline in an age-dependent fashion in the nervous system.”

The three gene expression studies which have investigated this issue in human brain found age- associated decreases in macroautophagy proteins, although the study by Guebel and Torres (2016) suggested differences between men and women for these parameters. However, confirmation that macroautophagy decreases in human brain during normal aging requires similar results with other methodologies. The lack of studies on age-associated changes in CMA in human brain specimens is a critical gap in the literature; unfortunately, the few CSF studies on this issue do not help to resolve the situation. Although the study by Baird et al. (2012) found the concentrations of three autophagy-related proteins to correlate with age, none of the proteins plays a major role in autophagy. The reports of an age-associated decrease in CSF HSPA8/hsc70 (Loeffler et al., 2016) and no change with normal aging in CSF lamp2 levels (Loeffler et al., 2018) provide no information about the effects of aging on specific autophagic mechanisms in the CNS. HSPA8/hsc70 participates in many processes in addition to CMA (Stricher et al., 2013; Liao and Tang, 2014) and measurement of total lamp2 is not specific for a particular autophagic mechanism. CSF studies are unlikely to be of value for determining the effects of aging on brain autophagy because (a) changes in the CSF concentration of a protein may not correlate with changes in its brain concentration, (b) the levels of autophagy-related proteins in CSF provide no information about their concentrations on lysosomal membranes; (c) it is unclear if the markers commonly used to assess changes in autophagic processes can be detected in CSF; and (d) measurement of the LC3-II/LC3-I ratio in the presence of inhibitors of lysosomal proteases or autophagosomal-lysosomal fusion is not possible in humans.

Some of these same issues raise concerns about the findings in the studies that have examined the effects of aging on brain autophagic processes in vertebrate models. Many of the studies on age-related changes in macroautophagy in experimental systems (Table 2) included measurements of total LC3, LC3-II, or the LC3-II/LC3-I ratio. The results in most of these studies are difficult to interpret with regard to autophagic flux because autophagic inhibitors were not included (exceptions: Kaushik et al., 2012; Rodríguez-Muela et al., 2013; Tashiro et al., 2014; Ott et al., 2016), and because they represent findings at only one time point. None of the studies on rat brain listed in Table 3 (effects of normal aging on CNS CMA in experimental systems) were performed on lysosome-enriched fractions, so the extent to which the reported levels of HSPA8/hsc70 reflected lysosomal concentrations of this protein is unknown. An inherent difficulty with comparing autophagy studies between different models is that basal autophagic activity in experimental animals can vary according to age, sex, and genetic background (Klionsky et al., 2016).

Comprehensive reviews of methods for assessing macroautophagy and CMA have recently been published (Patel and Cuervo, 2015; Klionsky et al., 2016). Appropriate procedures for investigating the effects of aging on these processes in the brain include transmission electron microscopy (TEM), western blot, immunohistochemical and immunofluorescent staining, and gene expression analyses. Combinations of these methods should be used (Klionsky et al., 2016). In addition to potential difficulties with data interpretation resulting from agonal changes and postmortem autolysis, studies on postmortem brain specimens provide only a “snapshot” of autophagic activity at a single time point, with no information about autophagic flux. TEM can detect and quantify morphological changes in autophagosomes, autolysosomes, amphisomes, and sequestered organelles (Eskelinen et al., 2011). Western blot is often used for examining changes in LC3-I and LC3-II levels (Tanida et al., 2005), but there are some caveats to the use of this procedure: (a) the concentration of LC3-I is higher than LC3-II in the brain, so LC3-II may be difficult to detect by western blot in crude homogenates (Chu et al., 2009; Yang et al., 2011); (b) LC3-I is more susceptible than LC3-II to degradation in SDS sample buffer or by exposure to freeze/thaw cycles; and (c) LC3-II levels measured by western blot should be compared to the levels of one or more loading control proteins rather than to LC3-I (Klionsky et al., 2016). Specific markers for CMA are presently limited to lamp2a and HSPA8/hsc70, but only the lysosomal membrane levels of these proteins correlate with CMA activity so their detection by Western blot or RT-PCR (Zhang et al., 2006; Jesus et al., 2013) should optimally be performed on lysosome-enriched fractions (Klionsky et al., 2016). Immunodetection of lamp2a should be done with antibodies which are specific for lamp2a (Patel and Cuervo, 2015).

Determining whether autophagic mechanisms decrease in the brain during normal aging has important clinical ramifications. Lifestyle modifications such as caloric restriction (Morselli et al., 2010; Rodríguez-Navarro and Cuervo, 2012; Rodríguez-Navarro et al., 2012) and exercise (He et al., 2012) have been shown to increase autophagic activity in experimental animals. Exercise reduces brain pathology in some mouse models of PD (Lau et al., 2011; Patki and Lau, 2011) and AD (Adlard et al., 2005; Nichol et al., 2008; Um et al., 2008), perhaps by induction of autophagy. Note, however, that a study in the P301S mouse model of tauopathy which examined the influence of long-term exercise on autophagy as well as tau pathology found reduced levels of full-length and hyperphosphorylated tau in the hippocampus and spinal cord despite no significant changes in the concentrations of LC3-II or p62/SQSTM1 (Ohia-Nwoko et al., 2014), suggesting that the neuroprotective effects of exercise in that study may have been due to factors other than induction of macroautophagy. Exercise can also exert neuroprotective effects by increasing synaptic protein levels, cell proliferation, and neurotrophic factors in the brain (Molteni et al., 2002; Naylor et al., 2005; Huang et al., 2006; Marlatt et al., 2012). In transgenic mouse models of AD, reversal of autophagic deficits reduces brain Aβ deposition and prevents learning and memory deficits (Caccamo et al., 2010; Spilman et al., 2010; Yang et al., 2011; Steele et al., 2013), and a reduction in α-synuclein aggregation and mitochondrial dysfunction has been found when autophagy is increased in experimental animal models of PD (reviewed by Moors et al., 2017). Taken together, these findings suggest that pharmacologic interventions and/or lifestyle alterations which increase brain autophagy might be useful for treatment and possibly even prevention of age-associated neurodegenerative disorders. The development of autophagy-inducing agents is an active field of research (reviewed by Levine et al., 2015; Vakifahmetoglu-Norberg et al., 2015). This approach is not without risk, because autophagy has been associated with increased tumor cell survival in some studies (reviewed by Gelino and Hansen, 2012). Further, in patients with impairments in autophagosomal-lysosomal fusion, such as AD patients with presenilin gene mutations (Lee J.Y. et al., 2010), treatment with autophagy- inducing agents might produce further accumulation of neurotoxic polyubiquitinated protein aggregates (Levine et al., 2015). Nevertheless, if autophagic mechanisms can be conclusively shown to decrease in the brain during normal aging, then interventions to prevent this decrease might reduce the risk for some age-related neurodegenerative disorders, as suggested by Cuervo and Wong (2014) and Loos et al. (2017).

## Author Contributions

DL planned and wrote the manuscript.

## Conflict of Interest Statement

The author declares that the research was conducted in the absence of any commercial or financial relationships that could be construed as a potential conflict of interest.
